# Models to predict the short-term survival of acute-on-chronic liver failure patients following liver transplantation

**DOI:** 10.1186/s12876-022-02164-6

**Published:** 2022-02-23

**Authors:** Min Yang, Bo Peng, Quan Zhuang, Junhui Li, Hong Liu, Ke Cheng, Yingzi Ming

**Affiliations:** Transplantation Center, Third Xiangya Hospital of Central South University, Changsha, Hunan People’s Republic of China

**Keywords:** ACLF, Machine-learning models, Liver transplantation, Prognosis, MELD

## Abstract

**Background:**

Acute-on-chronic liver failure (ACLF) is featured with rapid deterioration of chronic liver disease and poor short-term prognosis. Liver transplantation (LT) is recognized as the curative option for ACLF. However, there is no standard in the prediction of the short-term survival among ACLF patients following LT.

**Method:**

Preoperative data of 132 ACLF patients receiving LT at our center were investigated retrospectively. Cox regression was performed to determine the risk factors for short-term survival among ACLF patients following LT. Five conventional score systems (the MELD score, ABIC, CLIF-C OFs, CLIF-SOFAs and CLIF-C ACLFs) in forecasting short-term survival were estimated through the receiver operating characteristic (ROC). Four machine-learning (ML) models, including support vector machine (SVM), logistic regression (LR), multi-layer perceptron (MLP) and random forest (RF), were also established for short-term survival prediction.

**Results:**

Cox regression analysis demonstrated that creatinine (Cr) and international normalized ratio (INR) were the two independent predictors for short-term survival among ACLF patients following LT. The ROC curves showed that the area under the curve (AUC) ML models was much larger than that of conventional models in predicting short-term survival. Among conventional models the model for end stage liver disease (MELD) score had the highest AUC (0.704), while among ML models the RF model yielded the largest AUC (0.940).

**Conclusion:**

Compared with the traditional methods, the ML models showed good performance in the prediction of short-term prognosis among ACLF patients following LT and the RF model perform the best. It is promising to optimize organ allocation and promote transplant survival based on the prediction of ML models.

**Supplementary Information:**

The online version contains supplementary material available at 10.1186/s12876-022-02164-6.

## Background

Acute-on-chronic liver failure (ACLF) is a syndrome with acute exacerbation of chronic hepatopathy, characterized by intense systemic inflammation, multiple organ dysfunction, and poor prognosis [1–3]. Liver transplantation (LT) is regarded as the curative method for terminal liver diseases including ACLF [4, 5]. However, there is considerable discrepancy between the increasing ACLF patients waiting for LT and the shortage of available organ donors, and the 1-year post-transplantation mortality rate of ACLF still reaches approximately 20% [6]. Consequently it is necessary to establish the selection criteria of ACLF for LT, which may improve organ allocation and transplant outcome.

In previous studies, several scoring systems were applied to forecast the short-term outcome among ACLF patients. The model for end stage liver disease (MELD) score accurately evaluates the liver conditions and prognosis of terminal stage hepatopath, including ACLF, which had important implications for organ-allocation in emergency [7]. However, some studies have indicated a weak association between pre-transplant MELD score and post-transplant survival [8, 9]. The predictive value of other scores directed at ACLF, including the Chronic Liver Failure Consortium Organ Failure scores (CLIF-C OFs), CLIF sequential organ failure assessment scores (CLIF-SOFAs) and CLIF Consortium ACLF scores (CLIF-C ACLFs), has also been validated in ACLF patients [10–12]. However, few studies revealed these scores have good predictive value for short-term outcome in ACLF patients following LT. Therefore, it is essential to generate a new accurate prediction model for postoperative survival of ACLF following LT.

Machine learning (ML) leverages software algorithms to identify patterns in large data sets to establish predictive models more precisely than conventional methods. Machine learning algorithms can find novel patterns between variables and generate predictions by learning from multiple features simultaneously [13]. Several recent studies have indicated that ML models are useful for improving organ allocation and the transplant outcome after LT [14–18]. Previously our team had applied eight ML models for tacrolimus dose requirement post kidney transplantation [19]. The application of ML models is promising to forecast the short-term transplant survival of ACLF patients, which may contribute to organ allocation and benefit prognosis.

In the study, my team retrospectively analyzed ACLF patients receiving LT in our institution, and comparing the predictive value of conventional models and ML models for predicting 90-day posttransplant survival of these patients based on preoperative variables.

## Methods

### Study design

A retrospective study was designed to develop ML models to make a prediction for short-term prognosis in ACLF patients following LT. ACLF patients undergoing LT were enrolled from the Transplantation Center, Third Xiangya Hospital, Central South University between March, 2012 and December, 2019. The study protocol complied with the standards of the Declaration of Helsinki and obtained approval from the Institutional Review Board of Third Xiangya Hospital, Central South University (No. 2020-S398).

### Patients

Liver grafts with < 30% macrovesicular steatosis from donation after cardiac death (DCD) were all approved and distributed by China Organ Transplant Response System (COTRS). All recipients were administered Basiliximab as intraoperative induction therapy. The standard maintenance immunosuppressions consisted of calcineurin inhibitors (CNIs; tacrolimus or cyclosporin), mycophenolate mofetil or mycophenolate sodium (MMF), and prednisone. According to the criteria of the Asian Pacific Association for the Study of the Liver (APASL), ACLF was defined as “acute hepatic insult manifesting as jaundice (serum bilirubin > 5 mg/dL) and coagulopathy (INR > 1.5), complicated within 4 weeks by ascites and/or encephalopathy in a patient with previously diagnosed or undiagnosed chronic liver disease” [20]. Patient short-term survival was defined as 90 days' postoperative survival of ACLF patients. Preoperative variables including clinical characteristics and biochemical parameters were collected and analyzed. For patients with multiple biological data, the worst value during hospitalization in our department before LT was selected. All ACLF patients’ preoperative clinical data were used to calculate five conventional prediction formulas (the MELD score, ABIC, CLIF-C OFs, CLIF-SOFAs and CLIF-C ACLFs). The details of the formulas for the scores are shown in Additional file 1: Supplementary Table 1. All patients provided informed consent to participate in the study.

### Model building

Four ML classifiers were employed to predict 90-day post-transplant survival based on the patient’s preoperative variables: Support vector machine (SVM), logistic regression (LR), multilayer perceptron (MLP) and random forest (RF). The ML models were trained to build a prediction model using fivefold cross-validation and implemented via Python programming language (version 3.6) and Scikit-learn package (version 0.22) as previously reported [21]. In order to estimate the performance of different ML models, we applied k-fold cross-validation (with k = 5) and selected the good hyperparameters. In general, 132 patients were divided at random into five subgroups. A single subgroup is retained as the validation cohort for testing the final selected model while 4 subgroups are used as derivation cohort to create a formula. The average area under the curve (AUC) was computed by five independent runs. Finally, the algorithms were developed in the full data set of the eligible group.

According to the SVM model, the function was f(x) = SIGN (β_0_ + β_m_x_m_). As the value was 1, the patient was divided into the death group. As the value was − 1, the patient was divided into the survival group. According to the LR model, the function was f(x) = SIGMOID (β_0_ + β_m_x_m_). As the value was over 0.5, the patient was divided into the death group. As the value was no more than 0.5, the patient was divided into the survival group. The RF model was built with ten trees. Each tree in the forest is trained using a diverse portion of the database. Majority voting was performed to obtain the final predictions.

### Statistical analysis

Descriptive statistics are represented using means ± standard deviations (SD) or interquartile range (IQR) for continuous data and percentages for count data. Preoperative data were compared by Student’s t-test, the Pearson Chi-squared or Fisher exact test as appropriate. Kaplan–Meier (KM) survival curves and Cox regression analysis were made to analyze the risk factors for 90-day prognosis among ACLF patients following LT. The predictive power of conventional score systems for 90-day outcome was estimated by the receiver operating characteristic (ROC) curve. Results of *P* < 0.05 were thought to be significant and statistical calculations were performed using in SPSS version 22.0 (SPSS, Inc., Chicago, IL, USA) and GraphPad Prism 5 software (GraphPad Software, Inc., San Diego, CA).

## Results

### Basic characteristics

**Fig. 1:**
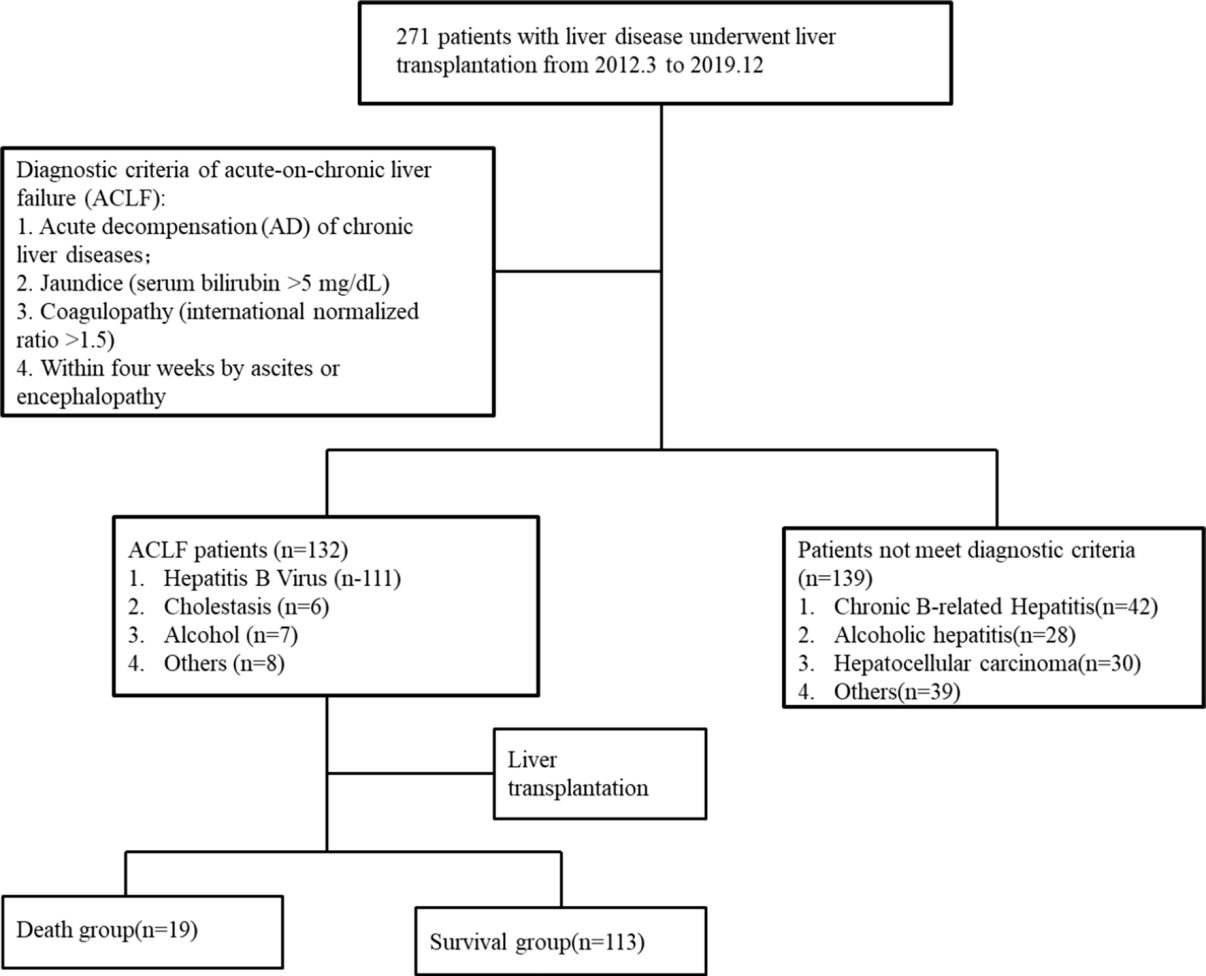
Flowchart of the search strategy and selection of studies for inclusion

A total of 271 patients accepted LT at our institution between March 1st, 2012 and December 31st, 2019, of which 132 patients achieving the APASL criteria of ACLF were eligible for this study. The flowchart for inclusion is shown in Fig. 1. All the patients included in the study also met the European Association for the Study of Liver (EASL)—Chronic Liver Failure (EASL-CLIF) Consortium ACLF criteria. The EASL-CLIF ACLF grades were shown in Table 1 and the detailed information was shown in Additional file 1: Supplementary Table 2. Nineteen ACLF patients (14.4%) died within 90 days after liver transplantation. The causes of death were upper gastrointestinal bleeding (n = 7, 36.8%), multiple organ dysfunction syndrome (MODS) (n = 6, 31.5%), severe pneumonia (n = 3, 15.8%), abdominal infection (n = 1, 5.3%), heart arrest (n = 1, 5.3%) and cerebral herniation (n = 1, 5.3%).

**Table 1:** Clinical characteristics of the patients

Characteristics	ALL(n = 132)	Survival(n = 113)	Death(n = 19)	*P* value
Age, mean ± SD (years)	45.92 ± 11.07	45.50 ± 11.04	48.37 ± 11.26	0.30
Etiologies, n(%)				0.29
HBV	111(84.1%)	96(85.0%)	15(78.9%)	
Cholestasis	6(4.5%)	4(3.5%)	2(10.5%)	
Alcohol	7(5.3%)	7(6.2%)	0(0%)	
Other	8(6.1%)	2(1.8%)	6(31.6%)	
Male, n(%)	108(81.8%)	94(83.2%)	14(73.7%)	0.51
ACLF Score				
CLIF-ACLF grade, n(%)				0.22
Grade 1	5(3.8%)	5(4.4%)	0(0%)	
Grade 2	91(68.9%)	80(70.8%)	11(57.9%)	
Grade 3	36(27.3%)	28(24.8%)	8(42.1%)	
TBILI, mean ± SD (μmol/L)	401.64 ± 198.29	404.00 ± 201.33	387.61 ± 183.55	0.74
Cr, mean ± SD (μmol/L)	84.71 ± 51.07	78.23 ± 37.09	123.26 ± 92.67	0.05
INR, mean ± SD	2.53 ± 1.22	2.44 ± 1.13	3.07 ± 1.58	0.11
HE, n(%)	29(22.0%)	23(20.4%)	6(31.6%)	0.27
PT, mean ± SD (s)	28.98 ± 13.95	28.28 ± 13.28	33.19 ± 17.24	0.25
Urea, mean ± SD (mmol/L)	7.83 ± 11.60	7.04 ± 11.20	12.54 ± 13.11	0.06
Alb, mean ± SD (g/L)	34.56 ± 5.68	34.43 ± 5.76	35.34 ± 5.30	0.52
DBiL, mean ± SD (μmol/L)	239.56 ± 131.65	244.93 ± 133.54	207.62 ± 117.93	0.26
Hb, mean ± SD (g/L)	105.07 ± 23.52	105.44 ± 23.19	102.90 ± 25.98	0.67
Plt, mean ± SD (10^3cells/μL)	92.53 ± 78.81	93.78 ± 82.80	85.05 ± 49.73	0.66
WBC, mean ± SD (10^3cells/μL)	8.06 ± 4.42	7.80 ± 4.19	9.59 ± 5.49	0.10
LYM, mean ± SD (10^3cells/μL)	1.09 ± 0.77	1.12 ± 0.78	0.92 ± 0.70	0.31
NEUT, mean ± SD (10^3cells/μL)	6.21 ± 3.80	5.95 ± 3.71	7.78 ± 4.05	0.05
Intensive Care Unit, n (%)	25(18.9%)	18(15.9%)	7(36.8%)	0.031
Renal replacement therapy, n (%)	7(5.3%)	3(2.7%)	4(21.1%)	0.001
Multidrug resistant organism infection, n (%)	3(2.3%)	2(1.8%)	1(5.3%)	0.344
Donor age, years	39.78 ± 13.25	40.50 ± 12.90	35.42 ± 14.82	0.51
Cause of death				0.09
Trauma	68(51.5%)	60(53.1%)	8(42.1%)	
Cerebrovascular accident (CVA)	49(37.1%)	43(38.1%)	6(31.6%)	
Other	15(11.4%)	10(8.8%)	5(26.3%)	
Donor risk index	1.40 ± 0.27	1.40 ± 0.23	1.38 ± 0.22	0.37
Steatosis				0.38
< 5%	38(28.8%)	30(26.5%)	8(42.1%)	
5% ~ 15%	70(53.0%)	62(54.9%)	8(42.1%)	
15% ~ 30%	24(18.2%)	21(18.6%)	3(15.8%)	
Cold ischemia time	7.52 ± 2.01	7.45 ± 1.97	7.89 ± 2.28	0.64

Tested by Student’s t-test or Pearson’s chi-squared (χ2) test; *SD* Standard Deviation; *HBV* Hepatitis B Virus; *TBiL* total bilirubin; *Cr* creatinine; *INR* International Normalized Ratio; *HE* hepatic encephalopathy; *PT* prothrombin time; *Alb* albumin; *DBiL* Direct Bilirubin; *Hb* hemoglobin; *Plt* platelet; *WBC* White Blood Cells; *LYM* lymphocyte count; *NEUT* neutrophil count

Comparison of clinical data between the survival group and the death group displayed no statistical difference in terms of age, sex, etiology, EASL-CLIF ACLF grade, total bilirubin (TBiL), creatinine (Cr), international normalized ratio (INR), hepatic encephalopathy (HE), prothrombin time (PT), urea, albumin, direct bilirubin (DBIL), hemoglobin, platelet count, white blood cells (WBC), neutrophil count (NEUT) and lymphocyte count (LYM) (Table 1). The characteristics of donors for the two groups also showed no significant difference, which was shown in the Additional file 1: Supplementary Table 3.

### Patient survival analysis

**Fig. 2:**
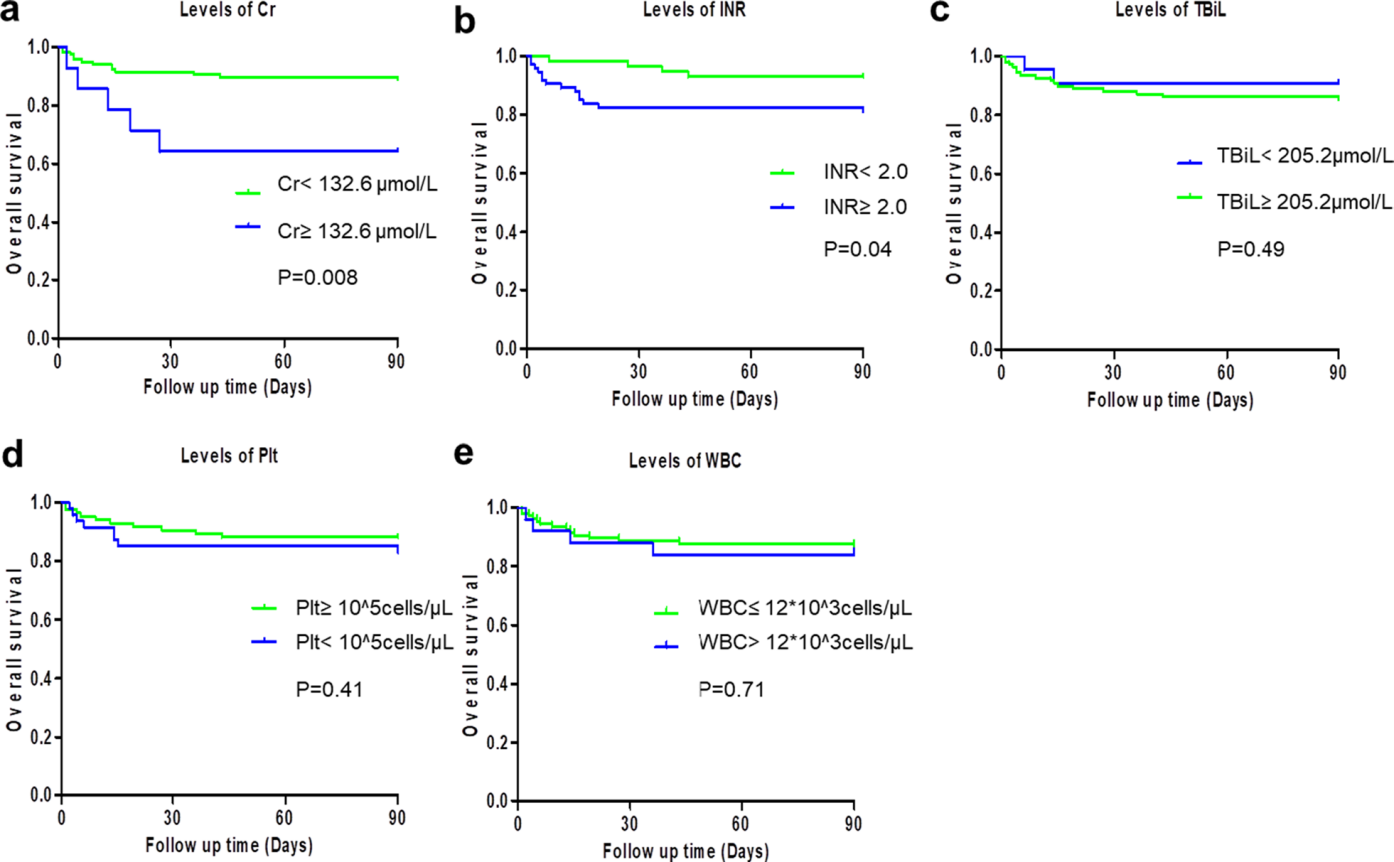
The overall survival curves of patients with the Kaplan–Meier estimator, tested with a log-rank test. **a** The overall survival curves of patients with different levels of Cr, *P* = 0.008. **b** The overall survival curves of patients with different levels of INR, *P* = 0.04. **c** The overall survival curves of patients with different levels of TBiL, *P* = 0.49. **d** The overall survival curves of patients with different levels of Plt, *P* = 0.41. **e** The overall survival curves of patients with different levels of WBC, *P* = 0.71. *Cr* creatinine; *INR* International Normalized Ratio; *TBiL* total bilirubin; *Plt* platelet; *WBC* White Blood Cells

Based on traditional formulae scores, the cut-off values of the important score-related parameters (Cr, INR, TBiL, Plt and WBC) were determined, which were applied for Kaplan–Meier (KM) survival analysis. The results indicated that the post-transplant mortality among ACLF patients was significantly associated with higher values of Cr (Cr ≥ 132. 6 μmol/L) and INR (INR ≥ 2.0) (*P* < 0.05). The differences among other factors were not statistically significant (Fig. 2).

The model of Cox regression was applied to identify the independent risk factors for short-term outcome. Univariate Cox regression analysis revealed that Cr (*P* = 0.001) and INR (*P* = 0.034) were poor prognostic indicators for ACLF patients following LT. Factors with *P* < 0.15 were further analyzed in multivariate cox regression. The results of multivariate analysis displayed that Cr (HR, 1.006; 95% CI, 1.001–1.011; *P* = 0.030) and INR (HR, 1.454; 95% CI, 1.100–1.921; *P* = 0.009) were independent prognostic markers of short-term outcome (Table 2).

**Table 2:** Univariate and multivariate Cox regression analyses of 90-day mortality

Characteristics	Univariate		Multivariate	
	*P* value	HR (95%CI)	*P* value	HR (95%CI)
Age, years	0.308	1.023(0.979 ~ 1.068)		
HBV	0.481	0.673(0.223 ~ 2.027)		
Male	0.354	0.617(0.222 ~ 1.714)		
TBILI, μmol/L	0.740	1.000(0.997 ~ 1.002)		
Cr, μmol/L	0.001	1.007(1.003 ~ 1.011)	0.030	1.006(1.001 ~ 1.011)
INR	0.034	1.347(1.023 ~ 1.773)	0.009	1.454(1.100 ~ 1.921)
HE	0.258	1.747(0.664 ~ 4.598)		
PT, s	0.152	1.019(0.993 ~ 1.045)		
Urea, mmol/L	0.071	1.019(0.998 ~ 1.040)	0.257	1.019(0.986 ~ 1.054)
Alb, g/L	0.507	1.026(0.950 ~ 1.109)		
DBiL, μmol/L	0.265	0.998(0.994 ~ 1.002)		
Hb, g/L	0.670	0.996(0.977 ~ 1.015)		
Plt, 10^3cells/μL	0.664	0.999(0.992 ~ 1.005)		
WBC, 10^3cells/μL	0.113	1.068(0.984 ~ 1.158)	0.284	0.743(0.431 ~ 1.279)
LYM, 10^3cells/μL	0.317	0.692(0.336 ~ 1.424)		
NEUT, 10^3cells/μL	0.060	1.095(0.996 ~ 1.203)	0.150	1.582(0.847 ~ 2.955)

Tested by univariate and multivariate Cox regression analysis; *HBV* Hepatitis B Virus; *TBiL* total bilirubin; *Cr* creatinine; *INR* International Normalized Ratio; *HE* hepatic encephalopathy; *PT* prothrombin time; *Alb* albumin; *DBiL* Direct Bilirubin; *Hb* hemoglobin; *Plt* platelet; *WBC* White Blood Cells; *LYM* lymphocyte count; *NEUT* neutrophil count

### Predictive value of conventional models

**Fig. 3:**
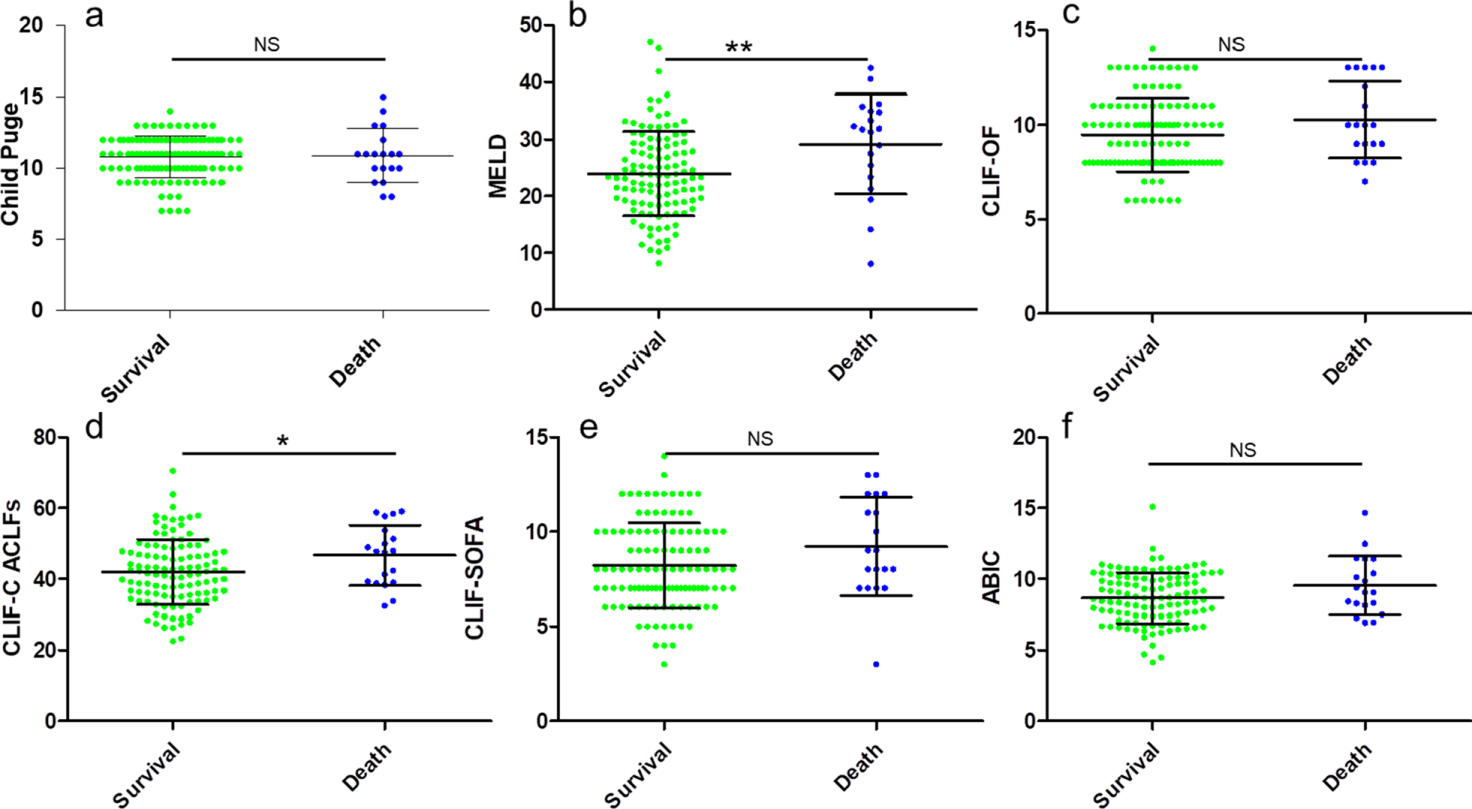
Scatter dot plot diagrams of the groups with conventional models to predict prognosis of ACLF following LT by Student’s t-test or Mann–Whitney U test. **a** Child puge between the survival group and death group, *P* > 0.05. **b** MELD score between the survival group and death group, *P* < 0.05. **c **CLIF-OF between the survival group and death group, *P* > 0.05. **d** CLIF-C ACLFs between the survival group and death group, *P* < 0.05. **e** CLIF-SOFA between the survival group and death group, *P* > 0.05. **f** ABIC between the survival group and death group, *P* > 0.05. The lines in the diagrams represent mean with SD. NS no significance *P < 0.05, **P < 0.01. *MELD* Model for end-stage liver disease; *CLIF-SOFA* Chronic liver failure-Sequential organ failure assessment; *CLIF-C OF* Chronic liver failure consortium Organ Failure score; *ABIC* age-bilirubin-international normalized ratio-creatinine

In comparison to those in the survival group, the scores of conventional models, including the MELD score, ABIC, CLIF-C OFs, CLIF-SOFAs, and CLIF-C ACLFs were higher in the death group. However, only MELD score (*P* = 0.01) and CLIF-C ACLFs (*P* = 0.04) showed significance between the survival group and death group (Fig. 3 and Table 3). According to the ROC analysis, the area under a receiver operating characteristics (AUROC) of MELDs (AUROC: 0.704) was higher than those of ABIC (AUROC: 0.607), CLIF-C OFs (AUROC: 0.606), CLIF-C ACLFs (AUROC: 0.653), and CLIF-SOFAs (AUROC: 0.633) for prediction of the 90-day outcome in ACLF patients following LT.

**Table 3:** Different scoring systems of the patients

Scoring systems	ALL(n = 132)	Survival(n = 113)	Death(n = 19)	*P* value
MELD Score, mean ± SD	24.69 ± 7.83	23.94 ± 7.44	29.13 ± 8.79	0.01
CLIF-OF, median (IQR)	9.5(8–11)	9(8–11)	10(9–13)	0.13
CLIF-C ACLFs, mean ± SD	42.57 ± 9.22	41.89 ± 9.19	46.62 ± 8.55	0.04
CLIF-SOFA, median (IQR)	8(7–10)	8(7–10)	9(7–12)	0.06
ABIC, mean ± SD	8.78 ± 1.85	8.66 ± 1.79	9.53 ± 2.06	0.06

Tested by Student’s t-test or Mann–Whitney U test; *SD* Standard Deviation; *IQR* interquartile range; *MELD* Model for end-stage liver disease; *CLIF-SOFA* Chronic liver failure-Sequential organ failure assessment; *CLIF-C OF* Chronic liver failure consortium Organ Failure score; *ABIC* age-bilirubin-international normalized ratio-creatinine

### Predictive value of ML models

**Fig. 4:**
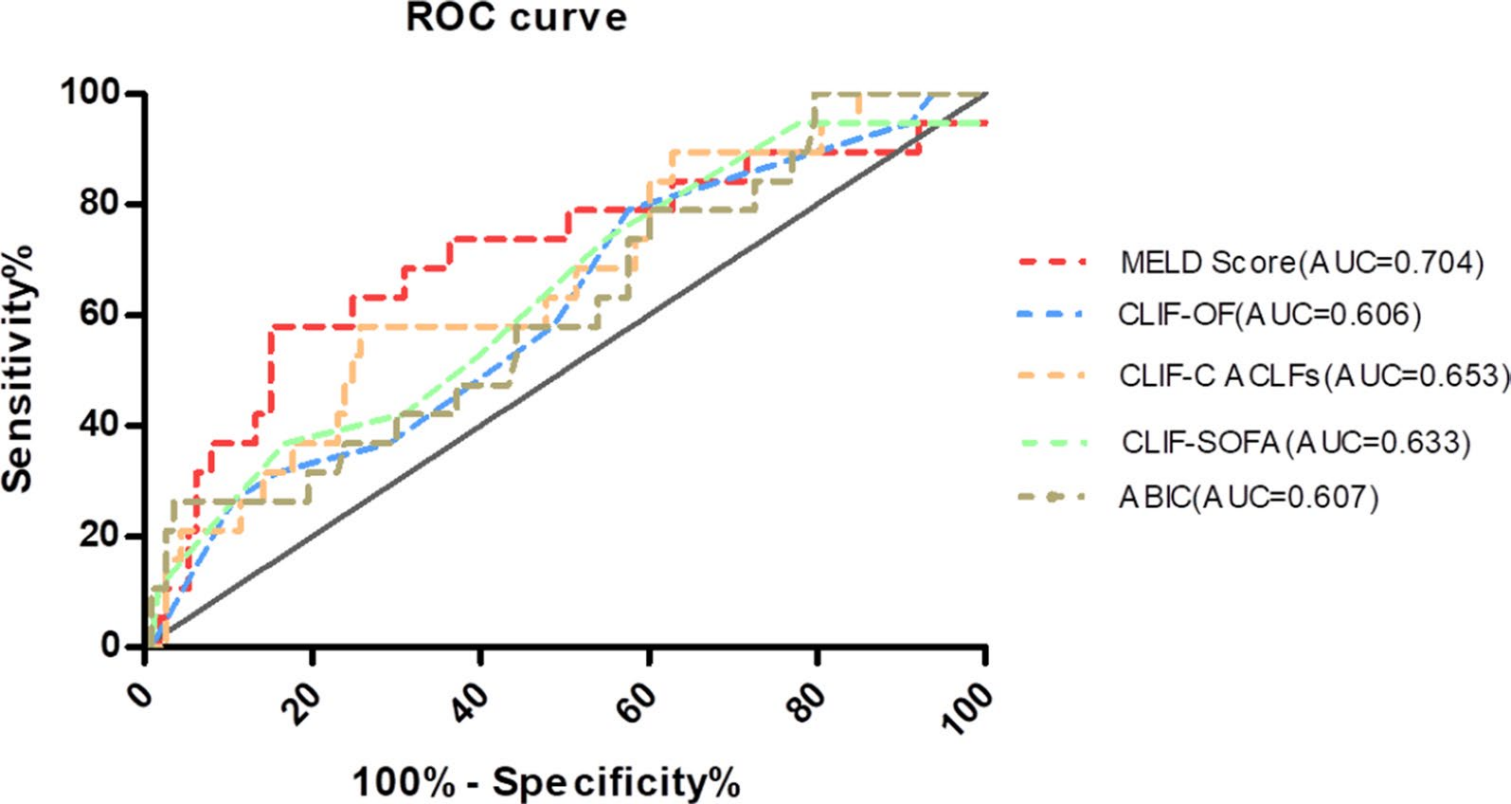
ROC curve comparison of conventional models to predict prognosis of ACLF following LT. *MELD* Model for end-stage liver disease; *CLIF-SOFA* Chronic liver failure-Sequential organ failure assessment; *CLIF-C OF* Chronic liver failure consortium Organ Failure score; *ABIC* age-bilirubin-international normalized ratio-creatinine

**Fig. 5:**
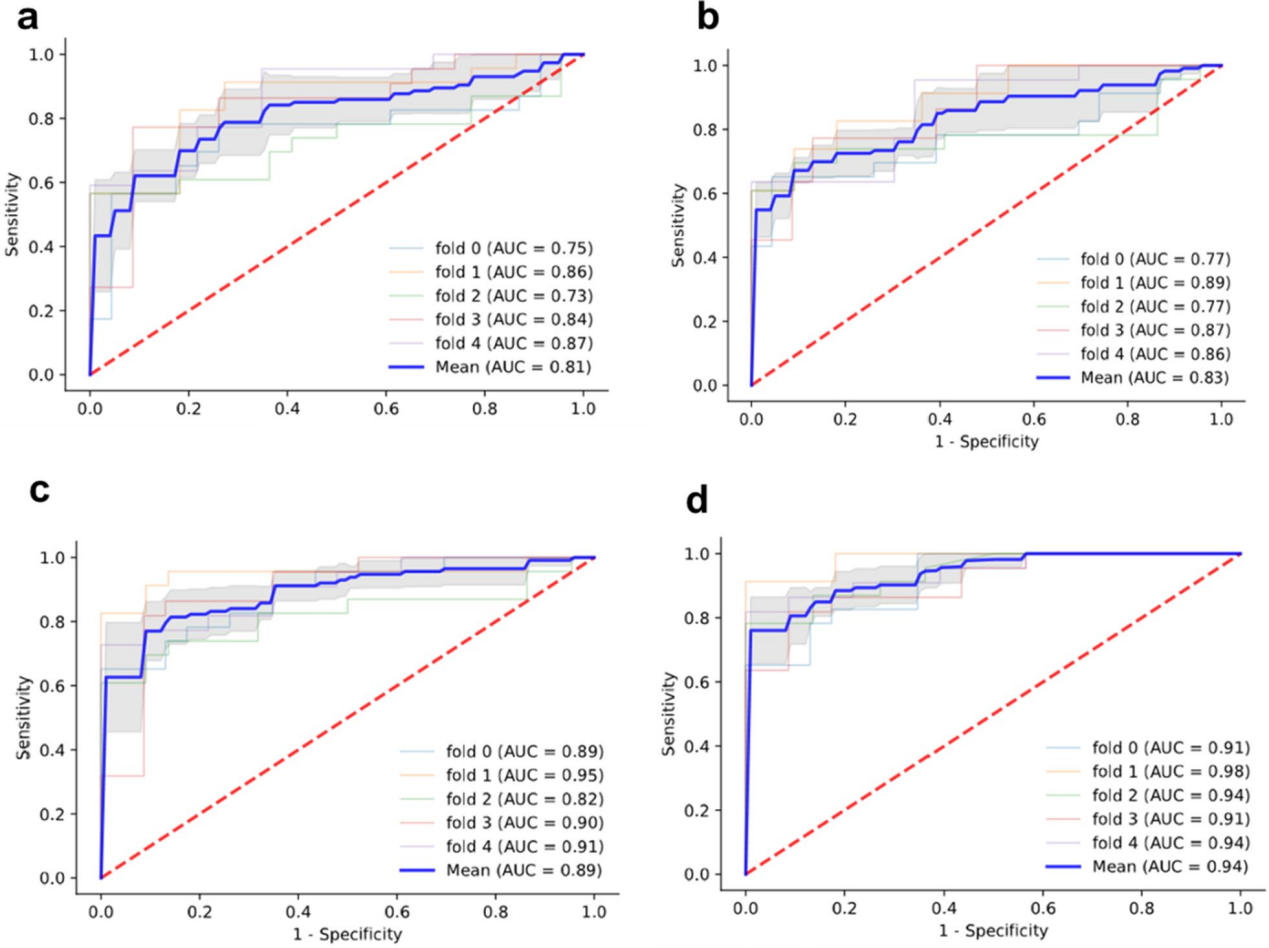
The ROC curves and average AUC of the machine learning models. K-fold cross validation (k = 5) was used to estimate and compare the performance of different machine learning models. After five rounds of training/validation rotation, the average AUC was calculated. **a** The support vector machine (SVM) model. **b** The logistic regression (LR) model. **c** The multi-layer perceptron (MLP) model. **d** The random forest (RF) model. *ROCcurve* receiver operating characteristic curve. *AUC* area under the curve

Four ML models (SVM, LR, MLP and RF) were trained and compared to improve the prediction performance. All ML models had good performance in terms of AUROC (Fig. 4), higher than those of conventional models. Among the ML models, the RF model had the highest AUROC of 0.94. The final result of RF model was derived from majority voting by the ten trees. The AUROCs of SVM, LR and MLP were 0.81, 0.83 and 0.89, respectively (Fig. 5). Cr, INR, etiology, DBiL, LYM and NEUT were chosen to develop the SVM and LR models. The coefficients of parameters in the models are described in Table 4. In the two models, the coefficients of Cr and INR are negative, indicating a negative correlation with the prognosis in ACLF patients following LT. The other parameters including etiology of liver disease, DBIL, LYM and NEUT, are positively associated with transplant outcome.

**Table 4:** The coefficients of the SVM and LR models

Parameters of models	SVM	LR
Cr	-4.24	-3.18
INR	-3.02	-2.12
HBV	1.43	0.76
DBiL	1.04	1.10
LYM	2.15	1.80
NEUT	-1.53	-1.43
Constant	0.29	0.31

*SVM* support vector machine; *LR* logistic regression; *Cr* creatinine; *INR* International Normalized Ratio; *HBV* Hepatitis B Virus; *DBiL* Direct Bilirubin; *LYM* lymphocyte count; *NEUT* neutrophil count

## Discussion

This study successfully established four ML models for forecasting the short-term survival of ACLF patients following LT. The ML model had better performance than the conventional models, and the RF model best predicted the short-term survival of ACLF patients following LT. ML algorithms could be a useful tool, facilitating better organ allocation and transplant outcomes.

Reportedly, there are several conventional models available to accurately estimate liver function and prognosis of patients with liver disease, such as Child–Pugh scores and the MELD. The allocation of donor Liver is based on the recipient’s MELD score, which has created a shift in donor liver allocation to the sickest recipients in order to minimize waiting list mortality [7, 22]. The MELD score is is widely used as a scoring system for organ allocation in liver transplantation and is the current standard prognostic tool for assessing 3- to 6-month survival in patients with hepatic failure [22]. However, ACLF has a unique clinical feature, and the predictor of MELD score for ACLF patients is not ideal. Liang Chen et al. exhibited that the ABIC score performed better than the MELD score in forecasting short-term survival among HBV-related ACLF patients [23]. Novel predictive assessment models (CLIF-C OFs, CLIF-SOFAs and CLIF-C ACLFs) have been developed and validated to forecast mortality in patients with ACLF, among which the CLIF-C ACLFs achieved better predictive accuracy than the MELD score [6].

Many previous researchers concluded that the MELD score and its exceptions had a limited ability to predict post-transplant mortality [24]. The MELD score could be correlated with post-transplant survival, but the pre-transplant MELD score appeared to have limited predictive ability [8]. No further studies showed that CLIF-C OFs, CLIF-SOFAs and CLIF-C ACLFs had good predictive value for short-term survival after LT. Our study indicated that the MELD score had a better AUROC (0.704) than other conventional models, but they all showed poor discrimination power in predicting postoperative survival.

Compared with traditional methods, ML algorithms utilize artificial intelligence to generate predictive models more precisely through the simultaneous detection of multidimensional parameters simultaneously [13]. The training set is employed to perform feature selection and parametric estimation, and the validation set is applied to assess the predictive power of the models. Furthermore, the model has the ability to self-evolve to adjust its structure when any errors are encountered. The models are promising in big data analysis, with the improvement of models performance using more data. Lau L et al. reported that RF model based on 15 donor and recipient variables had an excellent AUROC of 0.818 in forecasting the risk of liver graft failure in LT, thereby providing further evidence that the application of ML tools contributes to improving organ allocation decisions and transplant outcomes [14]. Hyung-Chul Lee et al. applied seven ML methods to predict acute kidney injury (AKI) after LT and the gradient boosting machine model exhibited the best performance with the AUROC of 0.90 [16]. Our team developed ML methods to confirm the relation between peripheral lymphocyte subsets and pneumonia among kidney transplant recipients for better individualized therapy [21]. Therefore, the ML technique could be a powerful and promising means in the evaluating of the prognosis of ACLF following LT. Our results showed that ML models had a better performance than conventional models, and RF model had the highest AUROC. These models were based on easily obtained parameters in clinic, making them practical in application.

Despite a growing number of LT performed in China over the years, there remains increasing discrepancy between the need for transplantation and the availability of donor organs. LT is considered a life-saving treatment for ACLF patients. The current policy for organ allocation in LT is to give priority to the sickest patients mostly using MELD score in ranking. It is difficult to consider whether the ACLF patient has a favorable prognosis following LT. Use of ML will dramatically enhance the efficiency of allocation of DCD organs for LT and contribute to maximal organ utilization. The selected preoperative variables in this study consisted of six parameters, namely, Cr, INR, etiology of liver disease, DBIL, LYM and NEUT. In our study, multivariate Cox regression modeling identified that Cr and INR were distinct prognostic factors of poor short-term survival in association with ACLF following LT. Cr is the most important component of the MELD score, which can objectively reflect the severity of chronic liver diseases and prioritize liver transplant candidates. Moreover, some data indicated that higher values of Cr were correlated with the poor prognoses of patients with liver diseases and LT recipients [22, 25]. First, pretransplant renal function was associated with renal insufficiency and increased short-term mortality following LT following LT [26, 27]. The occurrence of pretransplant renal function injury may result in an increase incidence of kidney failure and permanent kidney damage after LT [28]. Second, LT candidates with renal dysfunction have an increased risk of higher mortality risk from cardiovascular disease (CVD) after LT. Previous research indicated that pretransplant renal impairment was an independent indicator of post-LT CVD mortality among LT recipients [29]. The INR, a marker for coagulopathy, was one of the key components of both CLIF-C ACLFs and MELD, which emphasized the role of coagulopathy in forecasting the prognosis of patients with liver disease [30]. Our study demonstrated that the predominant etiology related to HBV (84.1%) of ACLF had a critical role in improving survival rates, which is consistent with the previous study [31]. The liver is the major organ of bilirubin metabolism, and hepatocytes are the only cells that can produce DBiL [32]. In ACLF patients, the higher direct bilirubin suggested moderate hepatocyte damage, consistent with the fact that DBiL was negatively correlated with prognosis in our study. Hypolymphemia and neutropenia are physiological responses to adverse stressful events, which often predict adverse outcomes of LT with ACLF. Bing-Yi Lin et al. illustrated that a high neutrophil–lymphocyte ratio indicated poor prognosis for ACLF after LT [6].

Some limitations of the study should be mentioned. Firstly, our study was a single center retrospective study. Secondly, the lack of clinical donor data may have slightly influenced the analysis results. Additionally, the small number of cases may have slightly affected the performance of the machine learning techniques. Future large-scale and multicenter are required to evaluate whether better organ allocation by machine learning algorithms could promote transplant survival.

## Conclusions

The ML models have good performance in predicting the short-term survival of ACLF patients following LT. The RF model best predicted the prognosis. With good predictive models, better organ allocation and clinical outcome can be achieved.

## Supplementary Information

**Additional file 1:** Supplementary Tables.

## Data Availability

The data that support the findings of this study are available from the corresponding author upon reasonable request.
