# Supplementary material for: Models to predict the short-term survival of acute-on-chronic liver failure patients following liver transplantation
Source: BMC Gastroenterol. 2022 Feb 23;22:80. doi: 10.1186/s12876-022-02164-6 (PMC8867783; doi:10.1186/s12876-022-02164-6)
Supplement: Supplementary file 1 — Additional file 1: Supplementary Tables. [file 12876_2022_2164_MOESM1_ESM.docx]

**Supplement Table 1: Formulas for scores to represent acute-on-chronic liver failure (ACLF)**

| **Scores** | **Formulas** | | | | | | |
| --- | --- | --- | --- | --- | --- | --- | --- |
| MELD score | 9.1×ln[creatinine(mg/dL)]+3.8×ln[bilirubin(mg/dL)]+11.2×ln(INR)+6.4×(etiology:0 if cholestatic or alcoholic, 1 otherwise). | | | | | | |
| ABIC score | (age × 0.1) + (bilirubin(mg/dL)× 0.08) + (creatine(mg/dL) × 0.3)+(INR × 0.8) | | | | | | |
| CLIF-SOFA | 0 | 1 | 2 | 3 | | 4 | |
| Liver(bilirubin, mg/dL) | ＜1.2 | ≥1.2 to ＜2.0 | ≥2.0 to ＜6.0 | ≥6.0 to ＜12.0 | | ≥12.0 | |
| Renal(creatine, mg/dL) | ＜1.2 | ≥1.2 to ＜2.0 | ≥2.0 to ＜3.5 | ≥3.5 to ＜5.0 or use of RRT | | ≥5.0 | |
| CNS(HE grade) | No HE | Ⅰ | Ⅱ | Ⅲ | | Ⅳ | |
| Coagulation(INR) | ＜1.1 | ≥1.1 to ＜1.25 | ≥1.25 to ＜1.5 | ≥1.5 to ＜2.5 | | ≥2.5 to platelet＜2*10^9/L | |
| Cardiovascular(hypotension) | MAP≥20 | MAP＜70 | Dopamine≤5 or dobutamine(any dose) or terlipressin | Dopamine＞5 or epi≤0.1 or norepi≤0.1 | | Dopamine＞15 or epi＞0.1 or norepi＞0.1 | |
| Respiration |  | | | | | | |
| PaO_2_/FiO_2_ | ＞400 | ＞300 to ≤400 | ＞200 to ≤300 | ＞100 to ≤200 | | | ≤100 |
| Or SpO_2_/FiO_2_ | ＞512 | ＞357 to ≤512 | ＞214 and ≤357 | ＞89 to ≤214 | | | ≤89 |
| CLIF-C OF |  | | | | | | |
| Liver(bilirubin, mg/dL) | 1 | 2 | 3 | |  | | |
| Renal(creatine, mg/dL) | ＜6 | ≥6 to ＜12 | ≥12 | |  | | |
| CNS(HE grade) | 0 | 1-2 | 3-4 | |  | | |
| Coagulation(INR) | ＜2.0 | ≥2.0 to ＜3.5 | ≥3.5 or ＜RRT | |  | | |
| Cardiovascular(hypotension) | MAP≥70 | MAP＜70 | Vasopressors | |  | | |
| Respiration |  |  |  | |  | | |
| PaO_2_/FiO_2_ | ＞300 | ≤300 and ＞200 | ≤200 | |  | | |
| Or SpO_2_/FiO_2_ | ＞357 | ＞214 to ≤357 | ≤214 | |  | | |
| CLIF-ACLFs | 10×[0.33×CLIF-C OFs+0.04×Age+0.63×ln(WBC count) ×2] | | | | | | |

Abbreviations: MELD: Model for end-stage liver disease; ABIC: age-bilirubin-international normalized ratio-creatinine; CLIF-SOFA: Chronic liver failure-Sequential organ failure assessment; CLIF-C OF: Chronic liver failure consortium Organ Failure score; INR: International normalized ratio; RRT: Renal replacement therapy; HE: Hepatic encephalopathy; MAP: Mean arterial pressure; PaO_2_: Partial pressure of arterial oxygen; FiO_2_: Fraction of inspired oxygen; SPO_2_: Pulse oximetric saturation.

**Supplementary Table 2: The CLIF-C OFs and 90-day mortality rate according to ACLF grades**

| ACLF grade | Number, n | CLIF-C OFs, median (IQR) | 90-day mortality after LT, n% |
| --- | --- | --- | --- |
| Grade 1 | 5 | 7(6-8.5) | 0% |
| Grade 2 | 91 | 8(8-10) | 11.0% |
| Grade 3 | 36 | 11(10-13) | 22.2% |

**Supplementary Table 3: Donor characteristics of the patients**

| Donor Characteristics | ALL(n=132) | Survival(n=113) | Death(n=19) | P value |
| --- | --- | --- | --- | --- |
| Donor age, years | 39.78±13.25 | 40.50±12.90 | 35.42±14.82 | 0.51 |
| Cause of Death |  |  |  | 0.09 |
| Trauma | 68(51.5%) | 60(53.1%) | 8(42.1%) |  |
| Cerebrovascular Accident (CVA) | 49(37.1%) | 43(38.1%) | 6(31.6%) |  |
| Other | 15(11.4%) | 10(8.8%) | 5(26.3%) |  |
| Donor risk index | 1.40±0.27 | 1.40±0.23 | 1.38±0.22 | 0.37 |
| Steatosis |  |  |  | 0.38 |
| <5% | 38(28.8%) | 30(26.5%) | 8(42.1%) |  |
| 5%~15% | 70(53.0%) | 62(54.9%) | 8(42.1%) |  |
| 15%~30% | 24(18.2%) | 21(18.6%) | 3(15.8%) |  |
| Cold ischemia time | 7.52±2.01 | 7.45±1.97 | 7.89±2.28 | 0.64 |
